# Timely initiation of new antenatal care contact model and its associated factors among pregnant women at Munessa health facilities, Ethiopia: a facility-based cross-sectional study

**DOI:** 10.3389/fmed.2025.1532041

**Published:** 2025-12-02

**Authors:** Efirem Hailu, Daniel Bekele, Getu Megersa

**Affiliations:** 1 Munessa Primary Hospital, Arsi Zone, Oromia, Munessa, Ethiopia; 2 Department of Midwifery, College of Health Sciences, Arsi University, Asella, Ethiopia

**Keywords:** antenatal care contact, knowledge, new model, pregnant women, timely initiation

## Abstract

**Background:**

The new antenatal care (ANC) contact model is recommended to be initiated within the first trimester of pregnancy, specifically before 12 weeks of gestation. The health of expectant mothers and the unborn children can be improved by early identification and treatment of preexisting and pregnancy-related conditions. However, in Ethiopia, there is limited evidence to support this. This study aimed to evaluate the extent of timely initiation, knowledge, attitude, and related aspects toward the new ANC contact model among pregnant women attending Munessa Health facilities.

**Methods:**

A cross-sectional study was carried out in a facility with 482 participants between April and May of 2024. The necessary sample was chosen by the use of a systematic sampling technique. Data were gathered from pregnant women who were visiting the prenatal care facility using an interviewer-administered questionnaire. Data were coded and entered using Epidata version 3.1 and analyzed by SPSS version 25. Binary and multivariable logistic regression analyses were used to identify possible determinants, and an odds ratio was used to measure the strength of associations at a *p*-value of <0.05.

**Results:**

The study revealed that 127 (26.5%) women initiated ANC on time. Generally, 215 (44.8%) pregnant women had a positive attitude toward and 83 (17.3%) of them had good knowledge of the new ANC contact model. Attending non-formal learning (AOR, 0.094, 95% CI 0.13, 0.56), being tested for pregnancy by urine (AOR, 6.4; 95% CI: 3.51, 12.03), having had no prior history of abortion (AOR = 0.47; 95% CI: 0.23, 0.97), and having no history of ANC complications (AOR = 0.076, 95% CI 0.03, 0.06) were significantly associated with the timely initiation of ANC contact.

**Conclusion:**

This study highlights the significance of exerting considerable effort to expand the coverage of timely ANC initiation. Consequently, raising mothers’ attitudes and level of awareness about ANC services and pregnancy hazard signals is crucial for expanding the coverage of timely ANC contact initiation.

## Introduction

Antenatal care (ANC) is the term used to describe the treatment that expectant mothers receive from trained medical professionals during their pregnancy. It protects the health of pregnant women and their fetuses during the pregnancy (1). In 2016, the World Health Organization (WHO) developed a new ANC model to improve the quality of prenatal care. It is advised that prior to the gestational age of 12 weeks, each pregnant woman should begin her first ANC contact (2, 3). This model suggests a minimum of 8 ANC contacts, with the first contact occurring in the first trimester of gestation followed by the second and third trimesters2 and 5 contacts, respectively (2). There are more maternal and fetal evaluations when ANC is started on time. Pregnant women who undergo these evaluations are more likely to have a successful pregnancy because these assessments can detect complications and improve communication between health providers and pregnant women, increasing the likelihood of positive pregnancy outcomes (4). Evidence shows that starting ANC within the first trimester facilitates the adoption of preventive measures, the early detection of diseases, and the provision of relevant and up-to-date information (5). It can also encourage the integration of clinical practices, the provision of psychosocial and emotional support, the reduction of pregnancy-related complications, and the elimination of health inequalities (6). Initiating ANC visits during the first trimester provides the best opportunity for conveying the key components of maternity healthcare services and increasing retention within the maternity care pathway (6, 7). Even though early ANC initiation has been shown to have benefits, many pregnant women begin ANC later than is recommended. Globally, the ANC initiation rate is 58.6%, however it varies per continent. The estimated rates of early ANC visits are 84.8% in nations with high incomes and 48.1% in low-income countries (4). Within the first trimester, 38.0% of ANC visits occur in sub-Saharan Africa (SSA). The percentage varies from 14.5% in Mozambique to 68.6% in Liberia (1, 8). In 2019, the Mini Ethiopian Demographic and Health Survey (EDHS) revealed that just 28% of women received their first antenatal care visit in the first trimester. This rate also varies across geographic regions (9). Previous research revealed regional and intraregional variations in the prevalence and related variables. Moreover, differences in the interpretation of when to initiate ANC were noted (6, 7). Considering the differences of ANC initiation of the previous model, WHO recommended early initiation of ANC attendance, a minimum number of ANC contacts and a minimum standard of care for the effective operationalization of ANC services (8). According to published research, a number of variables are linked to when the first ANC encounter occurs. These include maternal age, women’s level of education, marital status, ethnicity, and women’s involvement in decision making (3, 10). Though, there are some existing studies on timely initiation of the previous focused ANC visit in Ethiopia and in Oromia as well, no studies have been conducted considering the WHO new model ANC contact in Ethiopia. Furthermore, given the range of socioeconomic characteristics of the study population that may affect the timing of ANC beginning, there aren’t many studies exploring the timely initiation of the new ANC model and its associated factors in the study settings (Munessa district). Therefore, the findings of this study assist stakeholders and health officials in creating public health policies and strengthening social and familial support networks for expectant mothers in their communities. Thus, the objective of this study was to assess the magnitude of timely initiation of a new ANC contact model, knowledge, attitude and its associated factors among pregnant women at Munessa District health facilities, Ethiopia.

## Materials and methods

### Study design, area and period

A cross-sectional study design based on quantitative data was carried out between April and May 2024 among pregnant women receiving ANC at medical facilities in Munessa district, Oromia Region, Ethiopia. Munessa is one of the districts in Arsi Zone, Oromia Region, and is located 236 km from Addis Ababa, the capital city of Ethiopia. In the district, there are 7 health centers, 1 primary hospital, and more than 162 health professions. There are 166,539 people living in this woreda as of the 2007 national census, with a 2.5% annual growth rate. Of these, 82,559 are men and 83,980 are women. One primary hospital and seven health centers exist in the woreda. Currently, around 8,748 pregnant women visit the hospital and health centers for ANC services per year.

### Study source and study population

The source population consisted of all pregnant women who visited public ANC clinics in the Munessa area, while the study population consisted of all sampled pregnant women who visited public health facilities’ ANC clinics during the data collection period. Since it was believed that they would not be able to supply the required information, pregnant women with mental illnesses and those who were deaf or hard of hearing were not allowed to participate in the study.

### Sample size

We used the single population proportion formula to determine the sample size with the following assumptions: the proportion of timely initiation of new model ANC contact was taken from the study conducted in Nigeria (24%) (8) with a 95% confidence interval (CI) and a margin of error of 4%.


$$n=\frac{{\left(1.96\right)}^{2}\times 0.24\;(1-0.24)/{\left(0.04\right)}^{2}}{0.04^{2}}=438$$

Considering a non-response rate of 10%, the sample size was 482 women who initiated ANC contact.

### Sampling procedure

Using the simple random sampling method, four of the eight health facilities in the Munessa district were chosen. Before starting the study, the average monthly caseload of ANC visits at each chosen healthcare facility was evaluated. The population proportion to size for each chosen health facility was then used to determine the sample size for the study facilities. Finally, the study participants were questioned at each health facility either during the first or follow-up prenatal care. Consequently, *N*/*n* = 2 was used to determine the sampling frame and the total number of clients who received ANC from all institutions under study. A rigorous sampling strategy was used to interview every second client from all medical facilities. A random selection was made to interview the first client. As a result, the four chosen medical facilities had the following monthly client counts: Kersa Primary Hospital = 238; Kanchare Health Center = 64; Egoo Health Center = 83; and Kersa Health Center = 97.

### Data collection tool and quality control

A structured interviewer-administered questionnaire adapted from different literatures was used to collect the data (2, 3, 8). After being created in English, the tool was translated into Afaan Oromo, the local language, and then back into English by professionals to ensure consistency (reliability). Also, we used the Cronbach’s alpha to check the internal consistency of the multiple questions and scales of the tool (0.71 and 0.8 respectively). Four qualified data collectors with bachelor’s degrees in midwifery were recruited to fill the tools, and two midwives having master’s degree in clinical midwifery were recruited to supervise the data collection process. To avoid conflict of interest during the data collection process the data collectors and supervisors were recruited from the study sites. Data collectors and supervisors who took part in the pre-test and data collection received 2 days of training. To ensure the validity of the instrument, questions and ambiguities noted in the questionnaire were discussed after the pretest was completed. Following the conclusion of the pre-test, the questionnaire was finally revised. The supervisor and the lead investigator closely monitored and controlled the daily data collection. As the process progressed, data entry and code were verified. After data entry was complete, data cleaning was done.

### Variables

#### Dependent variable

The dependent variable is the timely initiation of ANC new model contact.

#### Independent variables

Sociodemographic factors such as age, educational status, marital status, occupation of respondents, monthly income, place of residence, religion, ethnicity, and husbands’ educational status and occupation, pregnancy-related variables (such as the number of deliveries, gravidity, and abortion history), types of pregnancy and means of recognizing pregnancy, and knowledge of women related to ANC visits are the independent variables.

### Operational definition

#### Timely initiation of new model ANC contact

This means that the first antenatal contact was before 12 weeks (during the first trimester) (2).

#### Knowledge regarding ANC service

It was assessed using 10 items, with a correct answer being given a score of “1” and an incorrect answer being given a score of “0.” Respondent’s overall knowledge was categorized, using a modified Bloom’s cut-off point, as good if the score was between 80 and 100%, moderate if the score was between 50 and 79%, and poor if the score was less than 50% (4).

#### Attitude on new ANC model

To measure the attitude level, 10 statements with 5-point Likert scale agreement options were used. Marks ranged from 1 to 5. There was a minimum of 10 and a maximum of 50 available points.

### Data analysis

After data were entered using Epi-Data version 3.1, it was exported to the Statistical Package for Social Science (SPSS) version 25.0 program for analysis. The main findings were compiled and presented using frequency and proportion. To choose candidate variables for the multivariable logistic regression, a bivariate logistic regression analysis was first used. The variables in the multivariable logistic regression model were those with a *p*-value of ≤ 0.25 in the bivariate logistic regression analysis. In order to adjust for potential confounders and identify the independent related factors of the outcome variable, it was carried out. An odds ratio was accepted at a 95% CI, and a *p*-value of < 0.05 was stated as statistically significant. Model fitness was checked by the Hosmer and Lomeshow goodness-of-fit tests (*p*-value = 0.773). Collinearity between independent variables was checked by taking the variance inflation factor (VIF) 10 as the cut point. In this case, VIF was 7.1. Finally, graphs, tables, and charts were used to present both the descriptive and analytical results accordingly.

### Ethical approval and clearance

The Institutional Review Board of Arsi University College of Health Sciences granted ethical permission (Number A/U/H/S/120/239/2015). Additionally, the healthcare facilities provided their legal consent. The goal and purpose of the study, as along with the freedom to discontinue participation at any moment, were explained to respondents. Written consent was then acquired from each respondent. For study participants under the age of 18, informed consent was acquired from a parent or guardian. Confidentiality was preserved by excluding personal identifiers such as names and addresses from the study.

## Results

### Sociodemographic characteristics

Out of 482 study samples only 480 (99.6% response rate) pregnant women responded in the study. The mean age of the respondents was 27.2 years (± 6.6 years), with approximately 37.7% of the women in the 25–29 age range. The respondents’ ethnic identity was Oromo (89.6%) and religion was Islam (62.1%). Nine out of ten pregnant women had completed primary education or above, and 99.4% of the participants were married. Regarding their occupation 56.1% pregnant women were housewives and 72.5% were rural residents, whereas 63.5% participants were located within a 30-min distance from the health facilities (Table 1).

**Table 1:** Sociodemographic characteristics of pregnant mothers at Munessa health facilities.

Variable	Category	Frequency	Percent
Age of participant in years	15–19	47	9.8
	20–24	133	27.7
	25–29	181	37.7
	30–39	97	20.2
	40–49	22	4.6
Marital status	Single	1	0.2
	Married	477	99.4
	Divorced	2	0.4
Religion	Orthodox	158	32.9
	Catholic	1	0.2
	Muslim	298	62.1
	Protestant	23	4.8
Ethnicity	Oromo	430	89.6
	Amhara	44	9.2
	Gurage	6	1.2
Mother’s education level	No education /never attended	70	14.6
	Primary	255	53.1
	Secondary	123	25.6
	Higher	32	6.7
Mother’s occupation	Housewife	271	56.4
	Employee	56	11.7
	Merchant	30	6.3
	Student	3	0.6
	Farmer	120	25.0
Husband’s occupation	Private employee	82	17.1
	Government employee	75	15.6
	Merchant	35	7.3
	Student	6	1.3
	Farmer	282	58.2
Area of residence	Rural	348	72.5
	Urban	132	27.5
Distance from ANC service (h)	Less than 30 min	305	63.5
	30 min-1 h	143	29.8
	More than 1 h	32	6.7
Family monthly income (Ethiopian birr)	1,651–3,200	7	1.5
	3,200–5,250	269	56
	5,251–7,800	179	37.3
	7,801–10,900	19	4.0
	≥10,900	6	1.3

### Obstetrics history of the respondents

Among pregnant mothers interviewed, the majority, i.e., 396 (82.5%), of them had more than one previous pregnancy and 99 (20.6%) had a history of abortions. Nearly 186 (38.7%) pregnant women had three or more children alive, and 92 (19.2%) pregnant women had a history of adverse pregnancy outcome. Ninety-two (19.2%) women had experienced pregnancy-related complications. In terms of current pregnancies, 293 (61%) women have planned to become pregnant. A urine test confirming pregnancy by measuring the HCG hormone revealed a value of 151 (31.5%).

### Timing of first ANC contact initiation

**Figure 1:**
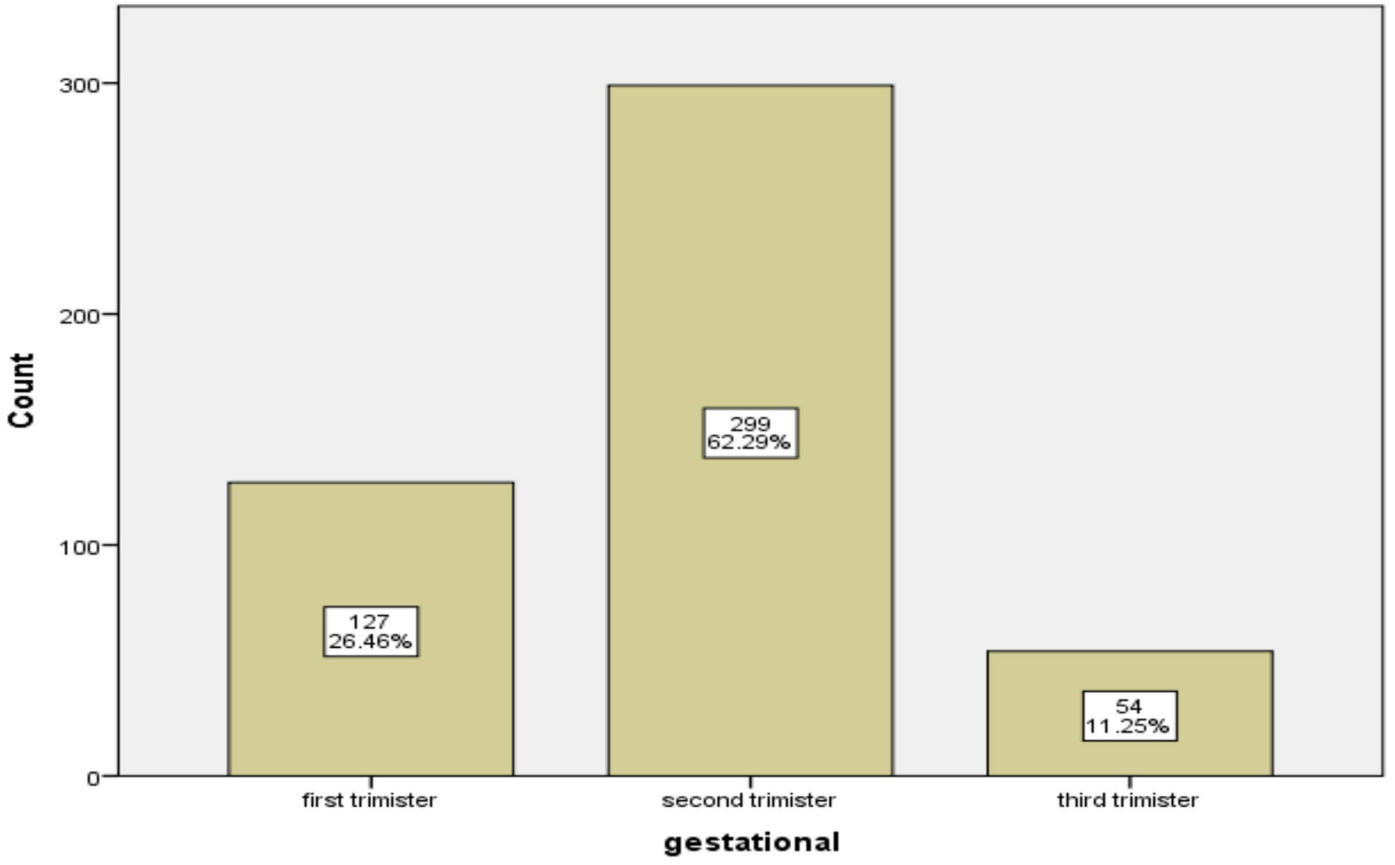
Distribution of first ANC contact among pregnant mothers in public health facilities of Munessa district (*n* = 480).

Nearly 127 (26.5%) pregnant women started their first ANC contact on time (less than 12 weeks of gestation). The first ANC appointment was scheduled between 7 and 40 weeks of pregnancy, and the average ANC visit time was 20.2 weeks (± 7.2 weeks). In the second trimester, 299 pregnant women (62.3%) began their first ANC initiation (Figure 1).

### Attitude toward the new ANC contact model

All respondents (100%) believe that all pregnant women should have ANC follow-up. Nearly 61.7% of the pregnant women perceived that eight ANC visits were better than four ANC visits for pregnancy and the fetus. A majority of respondents (98.4%) believe that husbands should be present during ANC contact, and 456 (95.1%) believed that ANC contact could reduce prenatal and postnatal problems. The majority of women (97.1%) strongly agree that iron and folic acid supplementation for all pregnant women is necessary, and 98.1% of the respondents perceived that having an ultrasound check-up is mandatory before 24 weeks. Overall, 215 (44.8%) pregnant women had a positive attitude toward ANC contact and timely ANC initiation, while 265 (55.2%) pregnant women had a negative attitude (Table 2).

**Table 2:** Attitudes of respondents toward the ANC contact model at Munesa.

ANC is highly important to the health of the mother	Strongly agree	472	98.3
	Agree	8	1.7
ANC is highly important to the health of the fetus	Strongly agree	406	84.6
	Agree	7	1.5
	Neutral	53	11.0
	Disagree	14	2.9
Antenatal booking is necessary for women before the 3rd month of pregnancy.	Strongly agree	323	67.3
	Agree	26	5.4
	Neutral	131	27.3
Pregnant women need to come for eight ANC contacts	Strongly agree	299	62.3
	Agree	20	4.2
	Neutral	160	33.3
	Disagree	1	0.2
Eight ANC contacts better than four ANC visits for pregnancy and fetus	Strongly agree	272	56.7
	Agree	24	5.0
	Neutral	161	33.3
	Disagree	23	4.8
Supplying iron and folic acid is good for the mother and fetus	Strongly agree	4.6	95.4
	Agree	8	1.7
	Neutral	14	2.9
All pregnant women should have ANC follow-up	Strongly agree	472	98.3
	Agree	8	1.7
Over all attitude	Positive attitude	215	44.8
	Negative attitude	265	55.2

### Knowledge toward the new ANC contact model

The definition of prenatal care was understood by nearly 73.3% of mothers, and 99.6% were aware of the significance of ANC for both the mother’s and the fetus’s health. However, only 24.5% of them were aware that ANC should begin within 12 weeks. Three hundred ninety-one (81.5%) pregnant women knew that ANC contact decreases maternal and fetal complications. Nearly 48.1% of women knew the correct time for an ultrasound scan, while 81% of them knew the recommended period of iron and folic acid (IFA) supplementation. Overall, in terms of participants’ knowledge of the new ANC contact model, 192 (40%) had good knowledge, 48 (10%) had moderate knowledge and 240 (50%) had poor knowledge level (Table 3).

**Table 3:** Knowledge of respondents on ANC contact model at Munessa health facilities.

Variable		Frequency	Percent
Definition of Antenatal care ANC	Yes	352	73.3
	No	128	26.7
ANC is highly important to the health of the mother and the fetus	Yes	478	99.6
	No	2	0.4
ANC booking before 12 weeks	Yes	118	24.5
	No	362	75.5
Number of ANC contacts (eight contacts)	Yes	32	6.7
	No	448	93.3
Pregnant women need to come for at least eight antenatal checks throughout her pregnancy	Yes	27	5.6
	No	453	94.4
ANC contact decreases antenatal and postnatal complications	Yes	391	81.5
	No	89	18.5
Husbands should be present during the ANC contact	Yes	448	93.3
	No	92	6.7
Service given in ANC contacts	Yes	89	18.5
	No	391	81.5
Gestational age of Ultrasound scan done less than 24 weeks	Yes	231	48.1
	No	249	51.9
Gestational age of IFA supplementation is less than 12 weeks	Yes	389	81.0
	No	91	19.0
Knowledge level toward ANC service	Good knowledge	192 (40%)	
	Moderate knowledge	48 (10%)	
	Poor knowledge	240 (50%)	

### Factors associated with the timely initiation of the new ANC contact model

Both the bivariate and multivariate logistic regression analyses, as shown in Table 4, revealed significant associations with mothers’ educational level, history of abortion, adverse pregnancy, prior pregnancy difficulties, and pregnancy confirmation methods. Women with no formal education and those with only primary education had 90.6 and 76%, respectively, lower odds of initiating ANC on time compared to women with higher education (AOR = 0.94, 95% CI: 0.13–0.67; and AOR = 0.24, 95% CI: 0.073–0.84). Respondents who confirmed their current pregnancy using a urine test were 6.4 times more likely to initiate ANC on time compared to those who confirmed it by a missed menstrual period (AOR = 6.4, 95% CI: 3.51–12.03). Conversely, pregnant women without a history of abortion were 53% less likely to initiate ANC early compared to those with a history of abortion (AOR = 0.47, 95% CI: 0.23–0.97). Mothers who experienced adverse effects during pregnancy were five times more likely to initiate ANC on time compared to those without such effects (AOR = 5.09, 95% CI: 2.58–10.03). In contrast, women with a history of normal previous pregnancy had 92.4% lower odds of timely ANC initiation compared to those who experienced complications in a previous pregnancy (AOR = 0.076, 95% CI: 0.03–0.16) (Table 4).

**Table 4:** Factors associated with timely new model ANC initiation at Munesa (*n* = 480).

Variable		Timing of ANC initiation		Odds ratio with 95% CI		*p*-value
		Yes	No	COR	AOR	
Educational status of the mother	No formal education	9	61	0.13 (0.04, 0.34)	0.94 (0.13, 0.67)*	0.019
	Primary	68	187	0.32 (0.15, 0.67)	0.24 (0.073, 0.84)*	0.026
	Secondary	36	87	0.36 (0.16, 0 0.80)	0.33 (0.10, 1.302)	0.079
	Higher	17	15	1	1	
Educational status of the husband	No formal education	4	39	0.14 (0.04, 0.44)	1.11 (0.11, 11.49)	0.99
	Primary	57	167	0.49 (0.298, 0.80)	1.62 (0.63, 4.175)	0.31
	Secondary	28	85	0.47 (0.26, 0.85)	0.91 (0.35, 2.38)	0.71
	Higher	41	59	1	1	
Residence	Rural	76	272	1	1	
	Urban	54	78	2.47 (1.61, 3.81)	1.36 (0.66, 2.33)	0.39
Distance from ANC service in hour	Less than 30 min	98	207	1	1	
	30 min–1 h	27	116	0.49 (0.30, 0.79)	0.98 (0.430, 2.25)	0.97
	More than 1 h	5	27	0.39 (0.146, 1.04)	0.86 (0.16, 4.50)	0.86
Media access	Yes	109	209	3.50 (2.095, 5.85)	1.38 (0.61, 3.13)	0.43
	No	21	141	1	1	
History of abortion	Yes	53	46	1	1	
	No	77	304	0.22 (0.13, 0.35)	0.47 (0.23, 0.97)*	0.04
Adverse effects of pregnancy	Yes	61	31	9.09 (5.49, 15.06)	5.09 (2.58, 10.03)*	0.001
	No	69	319	1	1	
Previous pregnancy complication	No	58	330	0.04 (0.02, 0.08)	0.076 (0.03, 0.16)*	0.001
	Yes	72	20	1	1	
Planned pregnancy	Planned	107	186	4.1 (2.49, 6.74)	1.73 (0.73, 4.06)	0.20
	Unplanned	23	164	1	1	
Received information on ANC	Yes	80	123	2.95 (1.94, 4.47)	1.41 (0.694, 2.89)	0.33
	No	50	227	1	1	
Methods of pregnancy confirmation	Missed period	55	274	1	1	
	By urine test	75	76	4.91 (3.19, 7.56)	6.42 (3.51, 12.03)*	0.001

*shows a significant of *p* < 0.05.

## Discussion

Timely initiation of ANC is a key strategy for meeting the new ANC model guidelines (the WHO’s 2016 recommendation) for a positive pregnancy experience (4). In this study, the prevalence of timely initiation of the new ANC contact model was observed to be 26.5%, which was similar to the rates reported in studies conducted in Hawasa (21.71%) (11) and Southwest Ethiopia, Illu Aba Bor zone (28.8%) (12). By contrast, a lower prevalence of timely initiation of ANC was observed in this study compared to those of studies conducted in Bahir Dar (48.6%) (6), Hosanna (34.3%) (4), Agaro (41.9%) (7), and Jimma (48%) cities of Ethiopia (10). It is possible that the greater percentages of timely ANC initiation resulted from the earlier research being carried out in a town district, where women are more knowledgeable about maternal health services. The fact that much of the earlier research concentrated on the ANC visit may possibly help to explain this discrepancy. The study revealed that most pregnant women had limited knowledge and a negative attitude toward the new ANC contact model and the correct timing of ANC initiation. Providing appropriate counseling on the recommended timing of ANC visits could encourage mothers to initiate care on time; when informed about the proper starting point, they are more likely to follow the guidance received. The results of this study also show that the prevalence of timely initiation of ANC services was lower compared to those in other countries, such as Benin (45.6%) (13), Ghana (68.0%) (14) and Uganda (50%) (15). This variation may be attributed to differences in socioeconomic status, access to services, and awareness levels. It could also be influenced by disparities in study settings and sample sizes. According to this study, ANC initiation at the WHO-recommended period (the first 12 weeks of pregnancy) was more common among women with secondary or higher education than among those without formal education. This may be explained by the fact that women who receive formal education are more likely to use appropriate maternity and child healthcare since they have a greater understanding of multiple aspects of health. This finding is also in line in with previous studies conducted in Hosana, Tigray, and Gahana (4, 14, 16). In addition, the greater likelihood of employment and higher income among educated women may provide them with better opportunities to access information and maternal health services compared to their less educated counterparts.

The likelihood of starting ANC on time was 6.4 times higher for respondents who used a urine test to establish their pregnancy than for those who relied on a missed menstrual period. This result aligns with an earlier study carried out in Southwest Ethiopia (7). This association may be due to urine tests being conducted at medical facilities, where women are often enrolled in ANC upon pregnancy confirmation. Early confirmation likely motivates women to initiate ANC sooner, as they become more aware of their pregnancy status. This study found that pregnant women without a history of abortion were 53% less likely to initiate ANC early compared to those with a prior abortion. This may be because women who have experienced previous pregnancy complications are more cautious and more likely to seek care earlier. This finding is consistent with results from the University of Gondar Hospital (17). This might be due to the fact that women who have experienced previous abortions may have a higher awareness of the importance of early ANC. They may understand the potential risks and complications associated with pregnancy and want to ensure they receive appropriate medical attention as soon as possible, and they may have learned from their previous experience that early detection and management of any potential issues can lead to better outcomes for both the mother and the baby (6). Furthermore, in this study, the odds of timely initiation of ANC were five times higher in mothers who had adverse effects of pregnancy as compared to those who had no adverse effects of pregnancy. This is supported by a study conducted in Ethiopia (18). The reason for this association might be that mothers who have experienced adverse effects and pregnancy complications during a previous pregnancy may have emotional and psychological factors that motivate them to seek timely initiation of ANC. Additionally, they may experience concerns or anxieties stemming from their previous pregnancy and seek to ensure they receive appropriate care and support during their current pregnancy.

### Limitations of the study

This study identified the magnitude and factors associated with timely initiation of the new ANC model; however, its cross-sectional design limits causal inference. Excluding women attending private health facilities affects generalizability, and a lack of similar studies in Ethiopia limits comparison. Despite these limitations, the study provides valuable insights into late ANC initiation and its associated factors in the study population.

## Conclusion

Most women in this study initiated their first ANC contact late. Key factors associated with delayed initiation included maternal education, history of abortion, pregnancy complications, and method of pregnancy confirmation. Overall, the finding that low maternal education and poor knowledge of the new WHO ANC contact schedule are significant barriers to timely ANC initiation has direct and specific implications for guiding health policy, intervention design, and health worker training in the Munessa district. Strengthening health education and community support systems is recommended, and the findings can inform policymakers and stakeholders in improving maternal health services by enhancing early ANC mode initiation.

## Data Availability

The raw data supporting the conclusions of this article will be made available by the authors, without undue reservation.
